# The Contribution of Long Noncoding RNAs to Worse Cognitive Function in Alzheimer's Disease

**DOI:** 10.1002/alz70855_103150

**Published:** 2025-12-23

**Authors:** Abdallah M Eteleeb

**Affiliations:** ^1^ Washington University in St. Louis, St. Louis, MO, USA

## Abstract

**Background:**

Long noncoding RNAs (lncRNAs) are increasingly recognized as key players in Alzheimer's disease (AD), influencing processes such as amyloid‐β production, tau hyperphosphorylation, synaptic plasticity, neuronal development, and neuroinflammation. However, their role in cognitive decline remains poorly understood. This study aims to elucidate how lncRNAs contribute to AD pathology using innovative multi‐omics integration and machine learning approaches.

**Method:**

To evaluate the utility of multi‐omics integration in uncovering molecular profiles of AD and associated lncRNAs, we employed machine learning to analyze multi‐modal omics data from various cortical regions and AD cohorts. This included data from the parietal cortex (*n* =  278, Knight ADRC), dorsolateral prefrontal cortex (*n* =  237, ROSMAP), and parahippocampal gyrus (*n* =  116, MSBB). We identified protein‐coding genes linked to AD and their interactions with dysregulated lncRNAs through gene regulatory networks, co‐expression, and protein‐protein interaction analyses. To explore the regulatory roles of lncRNAs in AD‐associated pathways, gene set enrichment and pathway analyses were performed.

**Result:**

Multi‐omics integration identified a distinct AD molecular profile linked to worse cognitive function, faster progression, shorter survival, severe neurodegeneration, astrogliosis, and dysregulated synapse‐related genes. RNA‐seq analyses revealed 177 lncRNAs, termed WCFALs (Worse Cognitive Function Associated lncRNAs), dysregulated across cohorts and brain regions. The most upregulated lncRNA, *WCFAL1*, showed a strong association with worse cognitive profiles and was consistently elevated in severe AD cases. Co‐expression and regulatory network analyses revealed a strong correlation (*r* = 0.78) between *WCFAL1* and *FOXN3*, a transcription factor known to regulate microglial function. Using RNA‐seq from iPSC‐derived cells and single‐nuclei data, FOXN3 was confirmed as predominantly expressed in microglia. Gene set enrichment and pathway analyses revealed WCFAL1's involvement in transcriptional binding, histone modification, and DNA regulation, indicating its potential role in regulating microglial activity through *FOXN3*. Protein interaction analysis showed *FOXN3* interacts with *SIN3A* (score = 0.95), a regulator of histone deacetylase complexes and amyloid‐beta.

**Conclusion:**

Multi‐omics analyses identified lncRNAs linked to worse cognitive function in AD. We hypothesize that *WCFAL1* regulates microglial activity via *FOXN3* or facilitates *FOXN3*‐*SIN3A* interaction. Future studies will validate this through *WCFAL1* overexpression or knockdown experiments.

